# A Novel Phosphopeptide Microarray Based Interactome Map in Breast Cancer Cells Reveals Phosphoprotein-GRB2 Cell Signaling Networks

**DOI:** 10.1371/journal.pone.0067634

**Published:** 2013-06-27

**Authors:** Srinivasan Krishnamoorthy, Zhonghua Liu, Ailing Hong, Ruijuan Zhu, Haosi Chen, Tongbin Li, Xiaochuan Zhou, Xiaolian Gao

**Affiliations:** 1 Department of Biology and Biochemistry, University of Houston, Houston, Texas, United States of America; 2 LC Sciences, Houston, Texas, United States of America; Biomedical Research Foundation, Academy of Athens, Greece

## Abstract

The architecture of cellular proteins connected to form signaling pathways in response to internal and external cues is much more complex than a group of simple protein-protein interactions. Post translational modifications on proteins (e.g., phosphorylation of serine, threonine and tyrosine residues on proteins) initiate many downstream signaling events leading to protein-protein interactions and subsequent activation of signaling cascades leading to cell proliferation, cell differentiation and cell death. As evidenced by a rapidly expanding mass spectrometry database demonstrating protein phosphorylation at specific motifs, there is currently a large gap in understanding the functional significance of phosphoproteins with respect to their specific protein connections in the signaling cascades. A comprehensive map that interconnects phospho-motifs in pathways will enable identification of nodal protein interactions that are sensitive signatures indicating a disease phenotype from the physiological hemostasis and provide clues into control of disease. Using a novel phosphopeptide microarray technology, we have mapped endogenous tyrosine-phosphoproteome interaction networks in breast cancer cells mediated by signaling adaptor protein GRB2, which transduces cellular responses downstream of several RTKs through the Ras-ERK signaling cascade. We have identified several previously reported motif specific interactions and novel interactions. The peptide microarray data indicate that various phospho-motifs on a single protein are differentially regulated in various cell types and shows global downregulation of phosphoprotein interactions specifically in cells with metastatic potential. The study has revealed novel phosphoprotein mediated signaling networks, which warrants further detailed analysis of the nodes of protein-protein interaction to uncover their biomarker or therapeutic potential.

## Introduction

Phosphoproteome analysis of breast mammary epithelial cells reveal multiple tyrosine phospho-motifs (pY) sites on proteins with large differences in phosphorylation stoichiometry which implies the possibility of functional significance of upregulated pY events in cellular communications [1]. Many such phospho-motif mediated protein interactions guide cellular responses of neoplastic transformation and metastasis. Phospho-protein enrichment coupled with high-throughput mass spectrometry based methods from various cell systems have led to catalogues of thousands of tyrosine phosphorylations on specific protein motifs that are still expanding rapidly [2], [3], [4], [5], [6], [7]. The phosphoproteome data indicate not only enormous complexity of cellular communication systems, but also the specificity of protein interactions in spatial and temporal dimensions. Understanding the biological significance of phospho-signaling networks will be of immense help in target refinement and drug development. Many anti-cancer drugs (especially tyrosine kinase inhibitors) induce undesirable side effects including cardiotoxicity, which significantly reduce the quality of life of cancer patients after chemotherapy [8], [9], [10]. Hence drugs developed to target phosphorylated motifs of a protein that induce specific cellular responses will be very effective with minimal off-target effects. Identification of phospho-protein based biomarkers is a sensible strategy for accurate prediction, diagnosis, prognosis, and risk classification of patients. To achieve this objective one must monitor protein interaction dynamics (upregulation or downregulation) mediated by multiple phospho-motifs on a high-throughput scale in order to distinguish physiological homeostasis from pathogenesis. Fabrication of integrated high throughput proteomic platforms to provide comprehensive maps of phospho-motif mediated interaction involving endogenous cellular proteins will help in a) identification of phosphoproteins that could serve as companion biomarkers for refining drug target specificity and b) development of protein profile signatures to rigorously test drug leads for their off targets before entering clinical trials to save time and money. Studies that underscore and justify the importance of targeting phosphoproteins in therapy bridge the gap between identification and understanding the presence of phosphorylation switches that regulate the biology of cancer progression and cellular responses to drugs [11], [12]. Understanding the functional significance of phospho-motifs on proteins that evoke the cellular response to attain metastatic potential is still an enigma. We hypothesize that specific nodes on the phosphoproteome-protein interactome could serve as signatures of pathway biology during normal and disease states and reveal clues for drug response. We have begun characterizing the phospho-tyrosine (pY) proteome by investigating the interconnection between phosphorylation sites on proteins and the corresponding phosphoprotein binding domain (PPBDs) containing proteins. Using a novel high density microfluidic µParaflo® PepArray technology (LC Sciences), we have generated a detailed map of endogenous RTK pathway phosphoproteome network mediated by GRB2 associated protein complexes that functions downstream of several RTK pathways in cultured cells from normal, tumor and metastatic breast tissues. Analysis of the peptide microarray data has not only validated phosphoprotein interactions reported by previous studies but also identified novel interactions that are worthy of follow up studies. The interaction dynamics as measured by the amount of GRB2 associated protein complex bound to the phospho-motif was sufficient to distinguish the cellular signature of a normal mammary epithelial cell (MCF10A) from that of breast cancer cells (MCF7, T47D and MDA-MB-231). We observed a generic down regulation of phospho-proteome-GRB2 interaction network in the metastatic tumor cell MDA-MB-231 compared to MCF7 and T47D tumor cells. The phospho-peptide microchip data is validated partly by published literature and by analysis of protein complexes in the cells used in the study by immunoprecipitation and western blotting. Our results demonstrate that various phospho-motifs on a single protein are differentially regulated and indicate the potential of targeting single phospho-motif for therapeutic intervention.

## Methods

### Source Peptides

Phosphopeptides were selected from various databases: PepCyber (P∼Pep), PhosphoSite, Swissprot and Phopho.ELM. PepCyber is an in-house protein interaction database (http://www.pepcyber.org/PPEP/) with a collection of phospho-protein motifs reported to interact with various SH2 domain containing proteins as determined either experimentally or by prediction models [13].

### Phosphopeptide Microarray Layout

PepArray Pro software developed in-house is a peptide microarray layout designer (http://www.pepcyber.org/PepArray/.) to suit a wide diversity of peptide layouts for the µParaflo® PepArray microchip platform [14], [15]. Based on published literature description of optimal binding motifs for SH2 domains [16], [17], [18], 6-mer peptide sequences were selected from target proteins starting with tyrosine followed by 5 residues from N to C terminal. For each pY peptide a corresponding control sequence was designed with alanine (A) substituted in place of phosphotyrosine (pY). Phosphopeptide source lists for SH2 and RTK arrays were arranged in to a structured layout using the option to create specific peptide panels following the steps of seed peptide generation, derived peptide generation and number of replicates selection. The control peptides serve as negative controls for technical validation of phosphopeptide specific protein binding. The sequence list provides all the information of each peptide probe (sequence, protein name, accession number etc) while the array layout depicts the peptide array design in a table format. The data layout sheet includes the reporter name (peptide ID with position of phosphotyrosine), actual sequence and the position of the peptide (with row and column information) on the chip. The layout files used for synthesis and data analysis of SH2 domain binding phosphopeptide array and the RTK peptide array are given as Supplementary tables S1 and S2 respectively.

### Reagents for Peptide Synthesis

The Fmoc and Boc amino acids, N-hydroxybenzotriazole (HOBt) and 2-(1H-benzotriazole-1-yl)-1,1,3,3-tetramethyluronium hexafluorophosphate (HBTU) were from GL Biochem (Shanghai Ltd) marketed by LC Sciences (Houston, TX). Fmoc-PEG-OH was ordered from Peptides International (Louisville, KY) and 3-aminopropyldiisopropylethoxysilane was from Gelest (Morrisville, PA). Bis (4-tetra-butylphenyl) iodonium triflate was purchased from Hampford Research (Strat-ford, CT). Other chemicals and organic solvents were ordered from Sigma-Aldrich (St. Louis, MO). All the chemicals were used without further purification unless otherwise stated.

### Chip Synthesis

The digital light gated microarray synthesizer consisted of a DNA synthesizer (Expedite 8909, PE Biosystems) as the automated reagent/solvent manifold and an optical unit with the same features as described previously [19]. The light source was 500W Hg lamp house (model 66033, Oriel Instruments) with a 405 nm filter. The microfluidic array was designed to contain 3968 reaction cells (128 rows ×31columns). The total reaction volume of the chip (1.2×2.0 cm^2^) is 10 µL and each cell can accommodate 0.2 nL of reaction volume (Fabricated at University of Michigan). The array was placed in a holder that was connected to the synthesizer in a way similar to connecting a CPG column for DNA synthesis. Peptide microarray synthesis was done on the µParaflo® Microchip System as reported before [15], [20], [21]. The peptide microarray synthesis is similar to conventional peptide synthesis using Boc chemistry except that a PGA (photo-generated acid) is being formed at selected reaction sites for N-Boc deprotection instead of TFA, allowing subsequent selective coupling of a Boc amino acid monomer at designated sites on a chip. Fmoc phosphotyrosine monomer was finally coupled to the N-terminal of each peptide probe by PGA, followed by removal of Fmoc with 20% piperidine in DMF. The microfluidic array system is optimized to synthesize phosphopeptides in isolated reaction cells in the order of picoliter volumes [15].

### Post Synthesis Treatment

After de-protection of peptides anchored on the chip, the chip was washed with ethanol and then washed with 25% acetonitrile overnight to make the peptide more soluble. The residual acetonitrile was removed by PBS washing for 2 hrs at room temperature. The surface blocking was conducted by incubation in blocking solution (1% BSA, 0.5% Gelatin, 0.05% TWEEN 20 in PBS; pH 6.8) at 4°C, overnight. ProQ staining on freshly synthesized phosphopeptide arrays was performed as part of peptide synthesis quality control procedure, to ensure uniform concentration of probes.

### Recombinant Proteins

Four recombinant proteins, Grb2 (Marligen Inc), Src (Invitrogen), BTK, and ZAP70 (Carna Biosciences) expressed in *E.coli* or insect cells were used in the SH2 domain substrate chip assay. BTK, Src and ZAP70 are full length proteins, where BTK and Src are fused with His tag but ZAP70 has no tag. The SH2 domain of GRB2 (93 amino acid residues) is a fusion with GST tag.

### Cell Culture

All the cell lines used in the study were purchased from ATCC and cultured according to the manufacturers’ protocol with the exception that low-glucose DMEM was used in the media.

### Cell Lysis and Protein Extraction

Cells were grown up to 80% confluence on 10 cm plates and washed with cold 1X PBS (4 mL) 4 times and lysed immediately in lysis buffer (20 mM Tris pH 7.0, 140 mM NaCl, 1% TX-100, 5% glycerol, 1% of phosphatase inhibitor cocktail (Roche), 2 mM sodium-ortho-vanadate and 1% protease inhibitor cocktail (Roche) for 1 hour at 4°C. The cells were centrifuged at 14K RPM for 20 min to remove the DNA and cellular debris. The protein supernatant was collected, filtered (0.2 um filter), aliquoted and stored at −80°C. The concentration was determined by Lowry method (DC assay; BioRad).

### Protein Binding Assay

For the binding assay, recombinant proteins and the total proteins from cells were diluted in protein binding buffer (PBB) containing 20 mM Tris (pH 7.0), 140 mM NaCl, 1% TX-100 and 5% glycerol. After equilibration with PBB, protein binding was done using either recombinant protein (200 ng/mL) or total protein from cell lysate (a total of 1 mg in 1 mL). The incubation conditions were 2 hrs at RT or overnight at 4°C for recombinant proteins and overnight binding at 4°C for total proteins from cell lysate. This was followed by primary antibody incubation (1 hr for recombinant protein at RT; overnight incubation at 4°C for cell lysate). The secondary antibody binding conditions are 1 hr at RT for both recombinant proteins and cell lysate. Between the steps of protein binding, primary antibody and the secondary antibody binding steps, the chips were washed with PBB for 1 hour at RT to remove excessive unbound reagents.

Details of antibodies used in the recombinant protein and cell lysate binding assays are as follows:

For recombinant protein binding assays the following florescent dye labeled primary antibodies were used: GRB2 (Anti-GST-Hylite 647); ZAP70 (Anti-ZAP70 Alexa 488); BTK (Anti-His-Hylite 647); SRC (Anti-His-Hylite 555).

For Cell lysate binding assays Rabbit-Anti-GRB2 and Mouse-Anti- SRC (Cell Signaling Technology, Beverley, MA) were used primary antibodies at a dilution of 1∶1000 and 1∶500 respectively. Secondary antibodies Alexa Fluor® 647 Donkey Anti-Rabbit IgG (H+L) and Alexa Fluor® 594 Goat Anti-Mouse IgG (H+L) (Life Technologies) were used as secondary antibody at a concentration of 25 ng/mL.

### Chip Imaging

The chip was scanned using Anon GenePix 4400A (Molecular devices) scanner using Genepixpro7 software. TIFF image files were further processed through Array-Pro Analyzer software and pixel density values were obtained as a text file (output data).

### Statistical Analysis

The pixel data was merged with the layout file using an In-house micro-array analysis program (Excel macros) and the data was then processed in multiple stages to obtain the final data that is background subtracted, replicates averaged and the absolute binding values of pY peptides obtained from subtracting each corresponding control peptide (A peptide). The output file consists of a single Excel file with multiple sheets: 1) raw data merged with the actual layout file, 2) processed data after background subtraction, 3) summary file with values for pY peptide and the corresponding control peptide and 4) a summary file that gives the list of statistically significant net signal (p = 0.01, p value of standard deviation ) for each pY peptide after subtracting the value of corresponding control peptide [22]. For heat map construction, the significant net signal value for each phosphopeptide interaction was subjected to log2 transformation and each peptide interaction was ranked using the Z score statistic to find the most significant interactions in each dataset and was compared across all the cell lines.

### Validation Assay for the Peptide Array Binding Data

Immunoprecipitation of protein endogenous complexes from cells with antibodies specific for GRB2, VEGFR1 pY 1213 and EGFR pY1092 was performed using 1 mg of total protein from cell lysate overnight at 4°C. About 20 uL of protein SEPHAROSE A/G Dynabeads (Life Technologies) were used to collect the antigen antibody complexes for about one hour. The antibodies used in immunoprecipitation assays are: GRB2 (anti-rabbit serum from Cell Signaling Technology, Beverley, MA) at a dilution of 1∶50; VEGFR1 pY 1213 antibody (R & D systems, Minneapolis, MN) at a dilution of (1∶100); EGFR pY1092 antibody (Assay Biotech, Sunnyvale, CA) at a dilution of 1∶100; PTPN11 pY584 (1∶100) and SHC1 pY427 (1∶100) from US Biological (Swampscott, MA). Immunoprecipitates were then washed three times with cold lysis buffer. Proteins in the IP complex were analyzed by resolving in PAGE gels and blotting with appropriate antibody. Primary antibodies used in the western blot analysis are GRB2 (anti-mouse monoclonal antibody (F-3 and H-9 clones) from Santa Cruz Biotechnology; 1∶100); PTPRA pY798 (Assay Biotech; 1∶1000); PTPN11 pY584 (1∶2000) and SHC1 pY427 (1∶500) from US Biological (Swampscott, MA); VEGFR1 pY 1213 antibody (R & D systems, Minneapolis, MN) at a dilution of (1∶500) and EGFR pY1092 antibody (Assay Biotech, Sunnyvale, CA) at a dilution of 1∶500. For loading controls we used anti-rabbit GAPDH (1∶1000) and anti- mouse β Actin (1∶1000) from Sigma. The secondary antibodies for rabbit primary antibodies are IRDye® 680RD Goat anti-Rabbit IgG (H+L) at a dilution of 1∶30000 and IRDye 800CW Goat anti-Rabbit IgG (H+L) at a dilution of 1∶20000 were used. For the mouse primary antibodies; IRDye® 680RD Goat anti-Mouse IgG (1∶30000) and IRDye 800CW Goat anti-Mouse IgG (1∶20000) were used. For Immunoprecipitation controls both rabbit IgG and mouse IgG (Sigma) were used @ 2 µg/1 mg of total protein. Signals were detected using the infrared dye conjugated secondary antibodies and signals detected using Odyssey IR image analyzer (LICOR Biosciences).

## Results

**Figure 1:**
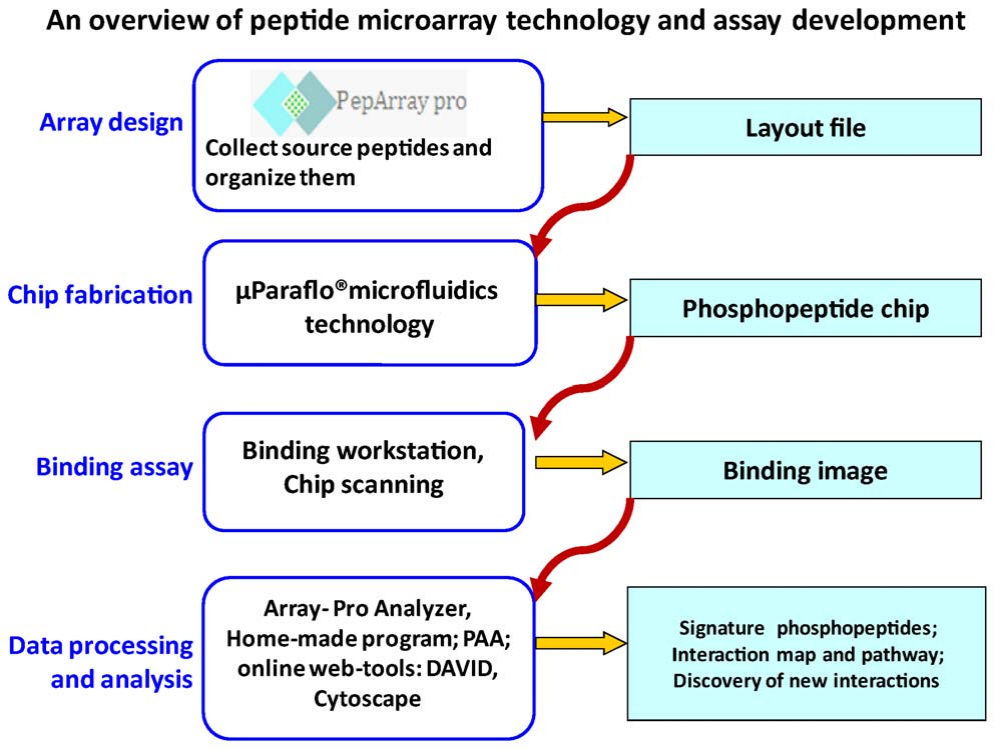
Work flow chart for the peptide microarray analysis of protein-protein interactions starting from peptide layout design to the final step of processed microarray data. The outcome of each technical step is depicted after the arrow. The process of layout file design followed by peptide chip synthesis, protein binding assay, imaging the microarray chip data, data processing and analysis are sequential and takes about a week to 10 days to complete one round of chip experiment.

The current efforts on understanding cell signaling networks using a systems biology based approach has led to the outflow of PTM databases from various labs with thousands of novel sites accumulated on a periodic basis (e.g. PhosphoSite). Discovery of such PTMs without the information of how are they interlinked in regulating protein-protein interactions as directed by signaling cues from inside and outside the cells will not reveal the biological function of these PTMs. Here we report a novel PTM (phosphotyrosine) based protein interactome map of breast cancer cells using a phosphopeptide microarray technology (µParaflo® PepArray) which integrates (i) a bioinformatics guided pathway-based peptide array design tool, (ii) *in situ* synthesis of phospho-tyrosine-peptides by photo generated acid (PGA) chemistry and by computer controlled digital lithography on microfluidic glass-silicon chip and (iii) a picoliter assay using a programmable microfluidic workstation. A collection of phosphomotifs from various public databases (PhosphoSitePlus, UniProtKB, Phospho.ELM, PhosphoPOINT, TiPD etc) is organized into a layout file to direct chip synthesis and data analysis. After the chip synthesis protein based assays are conducted and the resulting image file (consisting of all the fluorescent spots of phosphopeptide-protein interactions) is analyzed using a suite of in house software. The most significant interactions selected are analyzed using various online bioinformatics tools (like DAVID, KEGG, Cytoscape etc) to discover the signaling pathway biology. The various steps in peptide microarray profiling technology with technical details and the outcome are given in Figure 1.

In order to understand the application of this technology for studying tyrosine phosphorylation mediated protein-protein interactions on a global scale, we designed and developed a tyrosine phosphopeptide microarray and conducted assays using recombinant proteins and total proteins from cells. Here we present the results of these assays that demonstrated the utility of this platform for large scale protein interactome studies. Previously reported studies confirmed that the observed protein bindings on the peptide microarray are real interactions among proteins in cells. The technology opens the opportunity to refine the map of protein-protein interactions as precise interactions through specific PTMs and so a better means of identifying a well-defined protein-protein interaction involving a specific phosphoprotein. Targeting these phosphoproteins as drug targets will lead to an effective drug with lesser side effects (like cardiotoxicity) improving the quality of chemotherapy for cancer patients.

### Recombinant SH2 Domain Interaction on Phosphopeptide Microarray Reveals SH2 Domain Interaction Signature Consistent with Reported Studies

**Figure 2:**
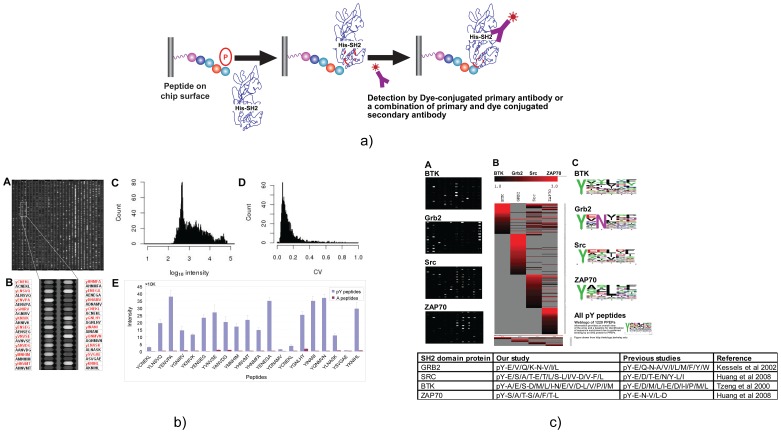
Details of recombinant protein binding assay on peptide microarray. **2(a)** Flow diagram showing the steps of recombinant protein binding assay on the pY peptide microarray synthesized on the chip surface. The recombinant protein containing an affinity tag (such as HIS, or GST) was applied to the microfluidic chip to be in contact with the pY peptide probes for about 1 hour. The specific interaction (binding) occurs between the protein and the pY peptide probe peptides based on the affinity of SH2 domain with phosphotyrosine residues on specific protein motifs. The binding was recognized by primary antibody (such as anti-HIS or anti-GST). Detection of the binding is through fluorescence dye (Hylite)-conjugated primary antibody. **2(b)** Recombinant protein binding on peptide array: Quality of binding image, data processing and analysis: The chip contained 3986 peptides synthesized *in situ* on a microfluidic chip surface containing 3,968 picoliter reaction wells with 1,227 peptides were tyrosine phosphorylated (pY-peptides) and 1227 corresponding control peptides (A-peptides) with technical controls for monitoring synthesis quality and protein binding assays. **A**. Binding image of GST-Grb2 SH2 domain on phosphopeptide chip: The array was probed with GST-tagged Grb2-SH2 domain and detected by antiGST-Hylite 647 conjugated antibody. Signals from proteins bound to phosphopeptides compared and respective control peptides wells were scored. **B.** Representative image of a small region from panel A. Peptide sequences (pY-peptides and A-peptides) are provided on either side of the image panel with the pY sequences marked in red and A-peptide sequences in black. The pY-peptide and corresponding A-peptide were synthesized at the two adjacent positions in the same column for conveniently visualizing pY specific binding. **C**. Histogram of the Grb2 binding (log_10_ scale) showing the distribution of binding intensities ranging from 500 to 60000 with a mean intensity around 1500. **D**. Histogram plot of the spot to spot CV (covariance of three replicate peptide-probes on chip) distribution of Grb2 binding: 3,695 (93%) spots have CVs less than 0.25, showing both peptide synthesis and Grb2 binding are uniform. **E**. Bar graph plot of the detected intensities of Grb2 SH2 domain binding shown as blue (**pY**-peptide probe signals) or brown (**A**-peptide probe signals). Grb2 SH2 domain binding affinity varied from 5 to 30 folds compared to control peptides. **2(c)** Comparison of peptide microarray SH2 domain binding motif consensus of this study (BTK, Grb2, Src and ZAP70) with those of previously published *in vitro* peptide binding results: Binding patterns and consensus sequences of four SH2 domain containing proteins (BTK, Grb2, Src and ZAP70). A. Binding images of four SH2 domains on the same region of four independent phosphopeptide chips. B. Heat map showing differential binding intensities (on Z value scale) of 4 SH2 containing proteins to related PPEPs on the chips. The pY probes with Z values ranked from 1.0–3.0 were plotted. C. Consensus sequences of binding sequences of 4 SH2 containing proteins. The consensus sequences were aligned by using WebLogo (version 2.8.2 http://weblogo.berkeley.edu/logo.cgi/). For each SH2 domain, the binding PPEPs with Z values greater than 1 were selected. The number of high confidence peptide probes selected to BTK, Grb2, Src and ZAP70 are 35, 133, 86 and 45, respectively.

In order to explore the utility of phosphopeptide arrays that we developed, we wanted to test if the chip platform could identify previously identified PTM mediated protein–protein interactions using conventional peptide based assays. We designed a peptide array consisting of 1226 tyrosine **p**hospho**pep**tides (PPEPs) representing phospho-motifs from 423 proteins from the PepCyber database [13] using the web based in-house interactive tool (PrpArray Pro). Using this layout as template the peptide microarrays were synthesized on the µParaflo® PepArray microchip system. The number of PPEPs per protein ranged from one to as high as 17 (EGFR). Since one PPEP may bind to multiple PPBDs and vice versa, a total of 2615 known interactions involving 101 PPBDs represented 33 pathways for cellular response and 13 disease pathways (Details of protein pathways covered by peptide probes representing each protein are given in Supplementary Table S3). We performed the binding assays using recombinant GST or His tagged SH2 domains from 4 different proteins (GRB2, SRC, BTK and ZAP70). Phosphopeptide-protein interaction signals were detected using respective florescent dye conjugated primary antibodies. The consistent high affinity binding signals from the chip image were selected after processing the image through a suite of in-house software programs. Each PPEP probe had two replicates with a corresponding control peptide (pY residue substituted by alanine) to identify SH2 domain interactions that are exclusive for tyrosine phosphorylation. Substitution of alanine is routine method to analyze bioactivity of a specific residue in the protein. When we were standardizing the assay, we started with control probes by substituting pY with A, Y and F. Even though the binding intensity data does not change with the substitution of A, Y or F for pY, the back ground was higher for both Y and F substitutions. So we decided to go with A substitution which gave a much cleaner background especially in recombinant protein binding assays. The cell lysate assay chips are not that pretty compared to recombinant proteins since we use total protein (mixture of at least 25000 proteins). The back ground spots appear only on the flow channels but the real protein binding signals in the reaction are generally very clean making the signal variations among replicates very minimal. Chip experiments were repeated twice per sample for each SH2 domain. A total of 160 PPEPs (BTK-45 probes; GRB2-56 probes; SRC-30 probes and ZAP70-55 probes) that showed consistently high binding affinity with any of the four SH2 domains were selected for total proteome screening from cell lysates. The binding affinity, as revealed by the signal intensity, ranged from 8000 to 60000 (saturated pixel density) with background signals in the range of 100–500 (Supplementary Table S4). For many known interactions, the SH2 domain affinity on phosphopeptides was at least three fold higher as compared to the control peptides and demonstrates the feasibility of identifying novel signature binding motifs on peptide arrays. A diagrammatic representation of binding assay (Figure 2a) in terms of image quality (A and B), signal distribution (C) and spot to spot variations (D) and a sample of data analysis (E) showing signal intensity of pY peptide specific binding of GRB2 mediated protein complexes compared to the control (Alanine substituted for pY) peptide are given in Figure 2b.

Analysis of high affinity phosphopeptide probes using WebLogo led to the identification of phospho-motif binding signatures for the four recombinant proteins used in the study. For high affinity binders with Grb2 SH2, we found that the consensus of phosphopeptide binding motif (pY-E/V/Q/K-N-V/I/L matched the consensus binding motif pY-E/Q-N-ψ (ψ - hydrophobic residues) as reported previously [17]. Based on our results and those from literatures, the asparagine residue at P+2 is essential for Grb2 SH2 binding, whereas that selectivity at P+1 and P+3 is apparent, but less stringent. These three amino acid positions at the C-terminal of pY are sufficient for optimal Grb2 SH2 binding with high affinity and specificity. We found similar match of consensus for BTK [18], SRC and ZAP70 protein binding assays [16]. A summary of binding consensus between the peptide microarray data and the data from conventional peptide binding assays for the four SH2 domain proteins used in the study is given in Figure 2c. Based on our experience using multiple recombinant proteins from multiple vendors, we see that the specificity of binding of recombinant proteins is lost with long term storage (more than 3 months at −80°C), so it is advisable to express them fresh and use immediately. This might be a potential issue to affect the quality of protein binding for the commercial available recombinant proteins unless they are custom made (made freshly for the user).

### Identification of Key Phosphoproteins that Mediate Endogenous GRB2 Protein Network in Breast Cancer Cells

**Figure 3:**
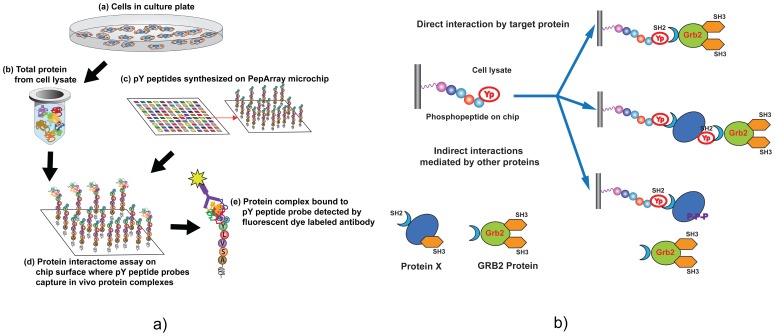
Phospho-PepArray analysis of signaling interactome from breast cancer cells. (a) Steps in phospho-motif binding assay of endogenous cellular protein complexes in cells: (a) cells are cultured on plates. (b) Total protein is isolated from cultured cells after cell lysis and cell lysate is applied to the phosphopeptides synthesized on (c) PepArray chip through microfluidics by circulation at 4°C overnight. (d) Antibody based detection is used to identify the protein of interest in these complexes. A general detection method is to stain the binding surface using anti-GRB2 antibody and a fluorescence dye conjugated secondary antibody such as Alexa. Based on *in vivo* substrate affinity of a specific phosphoprotein motif with binding domains on other cellular proteins, *in vivo* protein complexes, from the pool of non-denatured total proteins, are bound to respective phospho-peptides (pY) on the chip. **3(b)** Illustration of the possible peptide probe interaction with endogenous protein complexes from cells due to inter-protein interactions: Endogenous protein complexes containing SH2 domain in cell total proteome can bind to directly or indirectly to phosphopeptides (PPEPs) on the chip. For example GRB2, an SH2 domain containing protein can either directly bind to a phosphopeptide probe through the SH2 domain or can indirectly bind to the PPEP through interacting with the pY sites of a sandwich protein (Protein X) which is bound to PPEP directly through its SH2 domain. Another way of indirect interaction of GBR2 is through the SH3 domain (bind to poly-proline-rich regions) that might interact with ploy proline rich region of the sandwich protein bound to the pY peptide probe. The presence of GRB2 either by direct or indirect interaction with a pY protein trapped on the respective phosphopeptide probe on the chip results in an interaction signal detected by florescent conjugated secondary antibody.

After testing the feasibility of identifying SH2 domain-phosphoprotein interactions using recombinant proteins, we wanted to examine the feasibility of detecting *in vivo* protein-phosphoprotein interactions. Hence we developed a cell based total protein assay to detect endogenous phosphoprotein-protein interactions mediated by tyrosine phosphorylation events in cancer cells. The tentative hypothesis behind this expectation is that the concentration of tyrosine phosphopeptides is much higher (1–4 µM) than the concentration achievable *in vivo* (in the range of pM or fM) which will enable the phosphopeptide to trap the respective protein complex involving the phosphomotif. To test the hypothesis we designed a tyrosine phosphopeptide array with 160 high affinity SH2 domain binding phospho-motifs identified from the recombinant SH2 domain screens. Each peptide probe was replicated 10 times on the chip with a control peptide for each replicate. The phosphopeptide binding assay was performed on 4 different cell types derived from the non-tumorigenic epithelium (MCF10A) and ER positive tumor cells (MCF7, T47D) and a breast metastatic (MDA-MB231) cell line to detect and compare the GRB2-phosphoprotein interactome networks. The experimental scheme of the cell lysate binding assay (Figure 3a) and type of interactions expected from a complex mixture of total cellular proteins that might interact with each other through various phosphotyrosine binding domains and phosphotyrosine motifs (Figure 3b) are shown in Figure 3. The image file obtained after scanning the chip was measured by the absolute net signals of tyrosine phosphopeptide bound to GRB2 mediated protein complex detected by GRB2 protein specific primary antibody followed by the dye conjugated secondary antibody.

**Figure 4:**
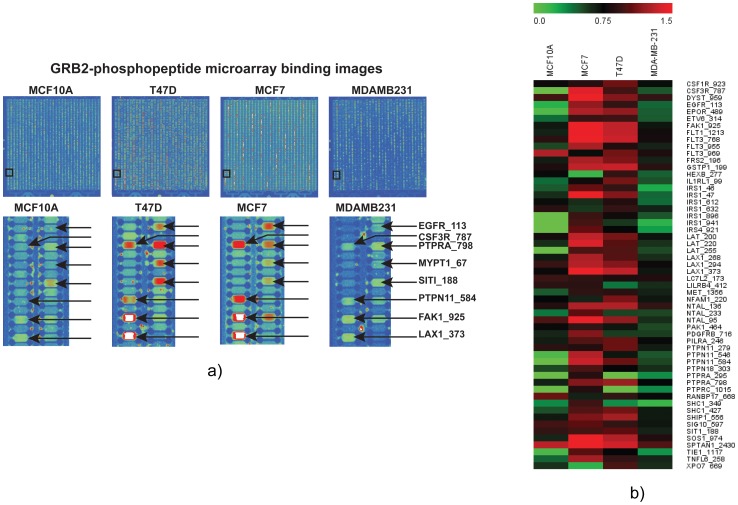
Highlights of the results of total cellular protein assay on pY peptide microarray. (**a**) Phosphopeptide microarray images showing spots of GRB2 association with specific phosphopeptide probes that represent phospho-motifs that are upregulated in ER positive breast tumor cells (MCF7 and T47D) compared to normal (MCF10A) or metastatic (MDA-MB231) cells that are ER negative. (**b**) Heatmap comparing high confidence GRB2 interactions from normal breast epithelial (MCF10A), breast tumor (MCF7, T47D) and metastatic (MDA-MB231) cell lines. The binding signals were normalized into Z values. The PPEPs with Z values ranked from 1.0–3.0 were selected to plot the heat map. The heat map clearly indicates upregulation of GRB2 interactome in tumor cells compared to normal or metastatic cells.

High-ranked phosphopeptide probes interacting with endogenous GRB2 from cell lysates were selected after a rigorous data processing using in-house software programs with appropriate statistical analysis of data normalization. A total of 57 phosphopeptide probes on 40 different proteins showed significant interaction differential (2–3 folds) that is sufficient to distinguish one cell type from another. Almost 70% of these interactions (40/57) were corroborated by previous studies and the rest are novel unreported interactions which proved the hypothesis of concentration mediated phosphopeptide-protein complex binding. Based on Pepcyber database predictions [13], 24 interactions are directly GRB2 mediated, 17 interactions are either direct or in a complex with other proteins and 15 interactions are indirect with one or many interacting sandwich proteins as we expected as a possible scenario in case of cell lysate with thousands of interacting proteins through different PTMs. (Table 1). Interestingly, the binding strength of these interactions can distinguish normal breast epithelial cells from ER positive breast tumor cells. In particular, several interactions were upregulated in non-metastatic cancer cells (MCF7, T47D) compared to normal (MCF10A) and metastatic cancer cells (MDA-MB231) as revealed by the absolute binding intensities (Table 2) and chip images (Figure 4a) and the heat map generated using Multi Experiment Viewer (MeV) (Figure 4b). Detailed molecular classification of phosphoproteins (representing the phosphopeptide probes) interacting with GRB2 revealed that the majority of these are associated with receptors (GF, T cell, B cell and cytokine signaling), adaptor proteins including tyrosine phosphatases, cytoskeletal regulators, nuclear transporters and RNA binding proteins (Supplementary Table S5). Almost all of these categories had phophoprotein-GBR2 interactions leading to a ras-Erk cascade that are reported to confer serum independent growth phenotype [23], ERK activation mediated proliferation and survival [24], [25].

**Table 1:** High confidence phospho-motif-GRB2 interactions from MCF10A, MCF7, T47D and MDA-MB231cells screened from peptide microarray analysis have been validated by previous studies as real interactions.

Phosphomotifs	Sequence (N′ to C′)	Swiss prot ID	Reported protein interactions from Pepcyber	Reported and novel GRB2 interactions
CSF3R_787	yENLWF	Q99062	SYK,SH3BP2	Hunter MG, et al. (2004), de Koning JP, et al. (1996)
DYST_959	yQNVLT	Q03001	GRB2	not reported
EPOR_489	yENSLI	P19235	LYN,SH3BP2,	Sulahian R, Cleaver O, Huang LJ (2009)
ETV6_314	yMNHIM	P41212	GRB2	Million, RP et al 2004
FAK1_925	yENVTG	Q05397	SRC,STAT3,SHC1,GRB2,FES,SH3BP2	Mitra SK, et al. (2006), Kaneda T, et al. (2008)
FLT1_1213	yVNAFK	P17948	PTPN11,GRB2,NCK1,PIK3R1	Ito N et al (1998, 2001)
FLT3_768	yENQKR	P36888	GRB2	Masson K, et al. (2009)
FLT3_955	yQNVDG	P36888	GRB2	Masson K, et al. (2009)
FLT3_969	yQNRRP	P36888	GRB2	Masson K, et al. (2009)
FRS2_196	yVNTTG	Q8WU20	GRB2	Hadari YR et al (1998)
GSTP1_199	yVNLPI	P09211	GRB2	Okamura T, et al. (2009)
IRS1_47	yENEKK	P35568	GRB2,SH3BP2	not reported
LAT_200	yVNVPE	O43561	GRAP2,GRAP,VAV1,PLCG1,GRB2,ZAP70,PIK3R1	Zhang W, et al. (1998, 2000), Malbec O, et al. (2004)
LAT_220	yVNVSQ	O43561	VAV1,GRB2,PLCG1,GRAP2,PIK3R1	Malbec O, et al. (2004)
LAX1_268	yVNMTG	Q8IWV1	GRB2,PIK3R1	not reported
LAX1_294	yENVPA	Q8IWV1	GRB2	not reported
LAX1_373	yENVLT	Q8IWV1	GRB2	Mayya V.et al. (2009)
NFAM1_220	yTALQR	Q8NET5	SYK,ZAP70	Yang J.et.al (2003). Ohtsuka M et al. (2004)
NTAL_136	yQNFSK	Q9GZY6	GRB2	Iwaki S, et al. (2008)
NTAL_95	yENVLI	Q9GZY6	GRB2	Iwaki S, et al. (2008)
PDGFRB_716	ySNALP	P09619	GRB2,GRB7	Arvidsson AK, et al. (1994)
PILRA_246	yENIRN	Q9UKJ1	PTPN11, PTPN6	not reported
PTPN11_279	yKNILP	Q06124	GRB2	Mitra S, et al (2008)
PTPN11_546	yTNIKY	Q06124	PTPN11,GRB2,FYN,SOCS3	Bennett AM, et al. (1994)
PTPN11_584	yENVGL	Q06124	PTPN11,GRB2,FYN	Araki T et al (2003)
PTPRA_798	yANFK	P18433	SRC,GRB2	Hao Q et al (2006); Chen M et al (2006)
SHC1_427	yVNIQN	P29353	GRB2	Ursini-Siegel J, et al. (2008); Patrussi L, et al. (2005)
SHIP1_556	yMNILR	Q92835	GRB2,INPP5D	CST curated data
SOS1_974	yQNQPY	Q07889	GRB2	not reproted
SPTAN1_2430	yQNLTR	Q13813	GRB2	Pighi C, et al. (2011), Rikova K, et al. (2007)
TNFL6_258	yVNVSE	P48023	GRB2	not reported
XPO7_669	yTALGR	Q9UIA9	PTPN11,PTPN6	not reported

A few novel interactions specifically the phosphorylation sites itself thus far are not reported.

**Table 2:** Data table showing GRB2 binding signal intensities of the high confidence phospho-motif-GRB2 interactions from MCF10A, MCF7, T47D and MDA-MB231cells.

Selected Phosphomotifs showing strong interaction with GRB2 on peptide microarray					
Reporter Name	MCF10A	MCF7	T47D	MDA-MB-231	Phosphoprotein-GRB2 reported
Molecular classification	Normalized GRB2 binding intensity				
**Receptor Tyrosine Kinases**					
CSF3R_787	23.64	39784.34	8264.28	1344.52	YES
FLT1_1213	1243.70	50329.84	42773.63	5265.04	YES
FLT3_768	3346.64	60294.52	47482.62	5622.40	YES
MET_1356	559.03	3902.83	5867.53	2945.71	YES
TIE1_1117	104.00	6881.89	3256.48	572.03	YES
**Other receptors**					
EPOR_489	90.03	20435.75	21415.85	1251.54	YES
PILRA_246	1347.84	6395.42	13985.79	4050.55	NO
PTPRA_798	1286.91	11705.29	25480.48	4155.81	YES
IL1RL1_99	426.15	2332.21	15882.67	1472.26	NO
**Signaling adaptors**					
SOS1_974	1133.47	56630.19	35405.88	6737.76	NO
FRS2_196	1299.14	18237.29	17453.82	2861.65	YES
IRS1_46	559.26	8414.52	6619.40	433.41	YES
IRS1_47	962.60	53236.66	18388.82	1093.66	NO
IRS1_896	40.37	4777.99	3742.82	1369.10	YES
IRS1_941	15.76	5455.87	1483.42	345.72	YES
IRS4_921	1.00	7686.77	3264.65	513.84	YES
SHC1_427	631.05	6357.72	15424.05	3265.95	YES
PTPN11_584	28.70	28289.57	13791.17	1636.27	YES
LAT_200	2429.98	25106.13	16252.66	3681.30	YES
LAT_220	972.14	36557.34	11143.63	2162.05	YES
LAT_255	48.83	5838.26	9974.29	1528.02	YES
LAX1_268	1409.30	19194.37	10462.40	2382.91	NO
LAX1_294	4367.68	22474.61	18570.96	4866.88	NO
LAX1_373	2000.16	62569.89	42557.02	3559.71	YES
NTAL_136	3019.42	21187.56	33979.91	9194.80	YES
NTAL_95	60.69	49842.24	22332.39	2257.07	YES
SHIP1_556	1472.04	10661.11	27023.16	3236.18	YES
**Cyotplasmic TKs**					
FAK1_925	1439.62	59992.23	41303.45	3586.70	YES
**Cytoskeletal Signaling**					
SPTA2_2430	23107.54	56317.28	50857.88	13934.16	YES
DYST_959	4886.17	56188.23	18325.11	8996.48	NO
MYPT1_68	104.58	4697.01	4038.31	1051.18	NO
**Ligand**					
TNFL6_258	487.91	17619.25	6887.68	2638.64	NO
**RNA Binding Protein**					
ETV6_314	426.82	4506.53	8044.14	1316.54	YES
**Enzymes**					
GSTP1_199	2594.76	25267.94	45756.98	7675.11	YES

The hip data are normalized taking average MCF10A chip signal as reference. A correction factor is obtained by dividing the average MCF10A chip signal with the average signals of MCF7, T47D and MDA-MB231data. The values for MCF7, T47D and MDA-MB231 are adjusted using the correction factor.

### Validation of Phosphopeptide Microarray Data by Immunoprecipitation of Protein Complexes and Western Analysis of Specific Phosphoproteins

**Figure 5:**
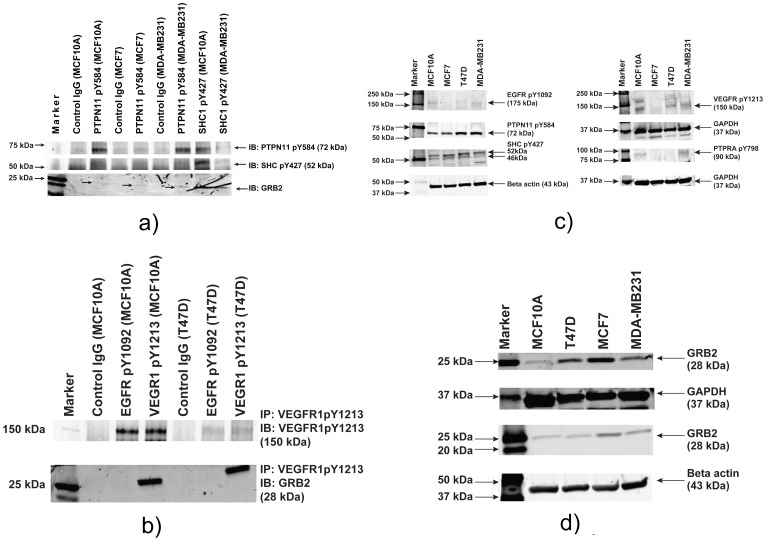
Validation and expression analyses of GRB2 and phosphoproteins in breast cancer cells that are associated by interactions on the phosphopeptide chip. **5a.** Immunoprecipitation and western analysis of endogenous protein complexes bound to phosphoproteins PTPN11pY584 and SHCpY527 from breast normal and cancer cells (MCF10A, MCF7 and MDA-MB231). The top and middle panel shows immunoprecipitated phosphospecific proteins PTPRA pY798 and PTPN11 pY584 while the bottom panel shows the presence of GRB2 (blotted with monoclonal anti- mouse GRB2 antibody) in immunoprecipitates of PTPN11 pY584 and SHC pY427. **5b.** Immunoprecipitation and western analysis of protein complexes bound to specific phosphoproteins in breast cancer cells (MCF10A and T47D) used in the study. Analysis of immunoprecipitates of phospho-specific antibody (VEGFR1 pY1213) and EGFR pY1092): Top panel shows the presence of phospho-VEGFR1 (pY1213) proteins in MCF10A (lanes 3 and 4) and T47D (lanes 6 and 7). The intensity of bands probably reflects the expression levels of VEGFR1. The bottom panel shows the presence of GRB2 in VEGFR1 immunoprecipitates but not in EGFR1 immunoprecipitates. In case of EGFR1 (pY1092) phosphoprotein the interaction of GRB2 may be indirect involving an unknown sandwich protein. **5c.** Analysis of level of expression of phosphoproteins used in the chip assay and cell based protein interaction studies. The expression levels were almost similar for PTPN11 pY584 and SHC pY427, but the difference was evident in case of EGFR pY1092, PTPRA pY798 and VEGFR pY1213 which is very minimal in MCF7 and T47D compared to MCF10A and MDA-MB231 but the GRB2 binding was much higher in chip assays with MCF7 and T47D. This indicates that the upregulation we see in chip assays might involve an increase in stoichiometry of tyrosine phosphorylation in tumor cells compared to the normal and metastatic cells. **5d.** The expression level of GRB2 indicated that the endogenous GRB2 is low abundant but the level of expression is upregulated in one of tumor cells (MCF7) followed by T47D which indicates that the upregulation of GRB2 binding in chip assays in tumor cells could be because of increased expression of GRB2 in these cells.

In order to confirm that the protein complexes from cell lysate pulled by phosphopeptide probes on the chip are in vivo interactions mediated by respective phosphoproteins, we conducted experiments to see if immunoprecipitation of GRB2 or phosphoproteins will reveal the interaction that we saw in the chip binding assays. Our attempts of immunoprecipitation of total GRB2 protein using anti-rabbit GRB2 antibody (CST) and analyze phosphoproteins bound to the complex had issues due to cross reaction of IgGs between GRB2 and the phosphoproteins (since almost all the phosphoproteins that are commercially available are raised in rabbits we had no choice but to use them). Since we had an option for the detection of GRB2 protein with mouse monoclonal antibodies (Santa Cruz Biotechnology Inc) we used phosphospecific antibody for a few proteins for which we saw GRB2 interaction on the peptide chip for immunoprecipitation and used mouse monoclonal GRB2 antibody to see the presence of GRB2 in the phosphoprotein complex. We selected very strong GRB2-phosphoprotein interactions based on bound GRB2 signals and did western analysis to detect specific phosphoproteins. After rigorous attempts using phosphospecific antibodies from several vendors (Cell Signaling technology Inc, Santa Cruz Biotechnology, Assay Biotech, US Biologicals, Abcam, Sigma and R&D systems etc), we were able to confirm GRB2 interactions with PTPN11pY584, SHCpY427, EGFRpY1092 and VEGFRpY1213 (Figures 5a and 5b). The western blotting signals of phosphoprotein bound to GRB2 did not match with the binding signals of GRB2 with the phosphopeptide probes. This may be due to (i) the quality of phosphospecific antibodies showing very weak binding and inability to reflect the in vivo differences at a very low concentration of phosphoproteins; (ii) loss of bound proteins in various stages of IP and western blots making it impossible to accurately detect the levels of endogenous protein interactions happening in the femtogram or attogram scale; (iii) What we see in the western blot is the endogenous GRB2 bound to a specific phosphomotif. But the lysate assay on the chip involves a complex scenario. On the chip we see the endogenous GRB2 bound to a protein complex wherein the phosphoprotein is one of the components. Since we cannot rule out the possibility of interacting with more than one protein in the complex, the discrepancy of binding intensity on the chip with that of the intensity on the western (IP followed by western analysis) is possible.

We also conducted protein expression analysis for some of the phosphoproteins that are used in the chip binding assay as well the immunoprecipitation assays to see as to how the expression level correlates with GRB2 binding to those phosphopeptides in the chip assays. Expression analyses of tyrosine phosphoproteins (40 ug of total proteins were blotted with respective phosphoprotein specific antibodies) also indicate differences between the cell lines used the study. Some of them (e.g. PTPRApY798, EGFRpY1092 and VEGFRpY1213) showed drastic differences in expression (Figure 5c). These observations further indicate that GRB2 binding to phosphopeptide probes does not reflect the differences in protein expression.

Expression level of endogenous GRB2 (40 ug of total proteins were blotted with GRB2 specific antibody) indicates that GRB2 is not abundantly expressed in the 4 cell lines used in the study but we did see differences in GRB2 expression levels among these cell lines (Figure 5d). Compared to MCF10A the expression levels of T47D and MDA-MB231 were 30 to 50% more than that of MCF10A whereas MCF7 was up by 130–150% than MCF10A. The binding intensity we see in chip experiments might be in part influenced by the differences in the expression levels of GRB2. But in chip experiments both T47D and MCF7 were almost equally upregulated than MCF10A and MDA-MB231. So in part the stoichiometry of tyrosine phosphorylation might be contributing to the binding of GRB2 on the chip assays.

Results of validation experiments in cells show that the GRB2–phosphopeptide interactions observed the phosphopeptide chips are real phosphoprotein-GRB2 protein interactions occurring in cells. With the availability of quality antibodies with higher affinity and specificity for phosphoproteins (which is the major impediment for the accurate mapping of protein-protein interactions so far), we can validate many interesting candidates we found in our chip assay and can refine protein-protein interaction mapping and improve the consistency to the level of using them as biomarkers to detect pathologic cellular responses.

### Comprehensive Mapping of pY Motif-GRB2 Networks in RTK Signaling

**Figure 6:**
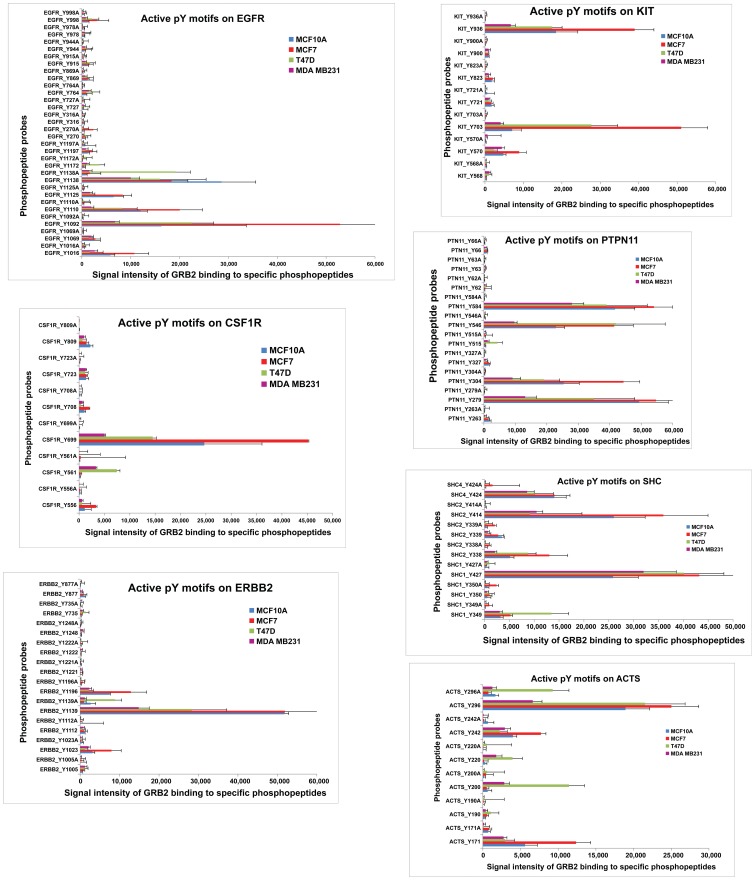
Mapping the strength of association of each tyrosine phosphomotif relevant to RTK pathways with GRB2 from normal breast cells and tumor cells. Comparative map of active pY motifs on various RTK signaling proteins in breast normal and cancer cells: MCF10A, MCF7, T47D and MDA-MB231. Each bar graph shows relative strengths of each of the tyrosine phospho-motif present in selected RTK pathway proteins interacting with GRB2 as measured by the signal intensity of GRB2 binding to Phosphopeptides (PPEPs) on microarray. The Y axis indicates all the phospho-motifs (source: Phosphosite) on the protein from all four cell lines compared with a control peptide (e.g.EGFR pY988 Vs EGFR Y988A). The X axis indicate intensity of GRB2 binding to each phosphopeptide probes (pY and A peptides). Each value is an average of three independent observations.

Encouraged by the results of SH2 domain containing phosphopeptide arrays, we have mapped active motifs from breast cancer cells using a phosphopeptide array that also included non-SH2 domain proteins. Array probes represented 49 RTKs, 28 cytoplasmic TKs, 32 signaling adaptor proteins and 82 other downstream signaling proteins including cytoskeletal interacters and Ras-Erk signaling mediators. Comparative analysis of GRB2 proteome interaction network in four different breast cell lines (MCF10A, MCF7, T47D and MDA-MB231) has revealed the whole spectrum of activation of various pY motifs on various RTK pathway proteins which represents missing links from the SH2 domain specific pY substrate array. Data analysis of 643 pY sites from 154 proteins (Supplementary table S6) revealed 27 RTKs, 29 cytoplasmic tyrosine kinases, 11 adaptor proteins, 4 cytoskeleton signaling related proteins and 3 Ras-ERK signaling proteins with specific tyrosine PTMs interacting with GRB2 containing protein complexes (supplementaryTable S7). Graphical representation of GRB2-pY motif interactions from selected proteins are shown in Figure 6. Consistent to the observation from SH2 domain binding phosphopeptide arrays, on a global scale, many GRB2-phosphoprotein interactions in MDA-MB231 are downregulated compared to tumor cells (MCF7 and T47D).

## Discussion

The outflow of mass spectrometry based methods has rapidly expanded the database of protein phosphorylation sites on several thousands of proteins. However, we can only understand their functional significance to evoke specific cellular responses in driving cellular signaling networks if we know their interconnections through specific protein-protein interactions. The PepArray technology platform enables simultaneous detection of multiple protein interactions initiated by specific phosphoprotein motifs on proteins representing various signaling pathways. We have developed and streamlined procedures for reproducibly detecting the endogenous GRB2-phosphoproteome –protein interactome network on a high-throughput scale. Using recombinant protein binding assays, we have demonstrated the ability to identify signature interactions involving specific phospho-motif on the chip surface. Using total proteins extracted from various cell types, we have shown that tyrosine phospho-motifs are part of protein complexes that interact directly or indirectly with GRB2. A set of these signature interactions clearly distinguished ER positive breast tumor cell lines (MCF7 and T47D) from the ER negative cell lines (MCF10A and MDA-MB231) evident form the following observations relevant to published literatures:

### GRB2 Interaction with MET and FAK could Provide a Marker to Distinguish Neoplastic Transformation from Metastasis

**Figure 7:**
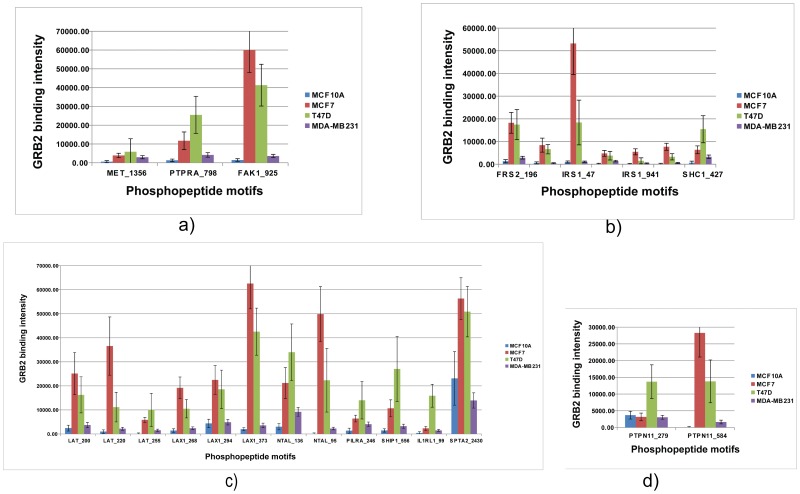
Graphical representation of dynamics of specific GRB2-tyrosine phosphopeptide interactions across all the four cell lines (MCF10A, MCF7, T47D and MDA-MB231). **7a.** Graph showing upregulation of GRB2 association with the MET receptor and FAK kinase. MCF7 and T47D are tumor cells and are ER positive while MCF10A and MDA-MB231 are ER negative. The observed decrease of GRB2 signals is consistent with previous studies reported. GRB2 interactions are significantly downregulated in metastatic cells (MDA-MB231). **7b.** Graph showing upregulation of GRB2 association with probes representing adaptor proteins IRS1 and SHC1 in R positive breast cancer cells. Based on reported literature, interactions of GRB2 with IRS1 pY motifs reveal potential cross talk between RTK and ER pathways. **7c.** Graph showing upregulation of GRB2 association with adaptor protein pY motifs in immune signaling (B cell and T cell) pathways: These interactions may indicate potential signaling events in tumor cells to overcome cellular immune defense mechanisms of the host cells. **7d.** Graph showing upregulation of GRB2 association with specific pY motifs on PTPN11 (SHP-2) protein: Down regulation of motif pY279 association compared to that of pY584 is with downregulation of ERK activation in tumor cells and suggesting alternate RTK pathways through pY584 for ERK activation. Each pY probe intensity value shown in the graph is an average of three replicates on the chip.

A handful of studies have demonstrated that cellular transformation by the Met oncoprotein requires pY1351 and pY1356 as functional GRB2 binding sites to regulate the process of neoplastic transformation and metastasis [26], [27], [28]. Mutation of the GRB2 docking site pY1356 uncouples GRB2 interaction and rescues the metastatic potential of cancer cells. It has been shown that the phosphatase receptor protein PTPRA in primary human keratinocytes reduces HGF-induced Met phosphorylation at pY1356 and inhibits downstream MEK1/2 and Erk activation [29]. Parallel to these observations, we see a drastically reduced interaction on METpY1356 and PTPRApY798 with GRB2 in metastatic cancer cells (MDA-MB231). FAKpY925 has been shown to be critical for association with paxillin and Erk activation leading to metastasis [30] and MAPK-associated angiogenesis mediated tumor progression through VEGFR [31]. Interestingly, in MDA-MB231 cells we see an inherent downregulation of FAKpY925-GRB2 association compared to the tumor cells (Figure 7a). These observations indicate that downregulation of GRB2 interaction with MET, PTPRA and FAK at specific phospho-motifs could predict if a neoplastic transformation can induce metastasis. A recent HT mass spectrometry based proteomic study on colon cancer [32] could not distinguish primary cancer from metastases based on global proteomic changes. But we show here that HT phosphoprotein interactome studies using peptide microarrays can unlock such intricate protein interaction dynamics.

### Interaction of GRB2 with RTK Signaling Adaptors Highlight Super Activation of Erk Through Different RTKs in Breast Cancer Cells

Of the several signaling adaptors identified in the screen (SHC, IRS1, IRS4 and FRS2), IRS1 is very active with 6 phospho-motifs (Figure 7b) out of which IRS1 (pY47) showed very strong association with GRB2. Parallel to several studies which implicate crosstalk between estrogens and insulin/IGF-I signaling in breast carcinogenesis, tumor cell proliferation, differentiation and survival [33], [34], [35], [36], our observation implies that IRS1pY47 interaction with GRB2 might be through SH2BP2 [13], a key regulator of ER alpha and IGF-1R signaling crosstalk in promoting cell proliferation and survival critical in ER positive (MCF7 and T47D) tumor cells. Sos1(pY974) predicted to interact with GRB2 [37] is an unreported novel interaction that we found upregulated in tumor cells might be a novel branch of Erk activation. An active motif on FRS2pY196 interacts with GRB2 to promote FGFR mediated ras-Erk activation [38]. GRB2 interaction with SHC1 (pY439 and pY427) has been shown in T cells to induce Ras-Erk signaling and CD69 leading to tumor cell survival and promoting tumor vascularization [39]. Taken together activation of multiple phospho-motifs on multiple adaptors confirms a quantitative signaling effect channeled for super-activation of ERK conferring multiplication and survival advantages in tumor cells.

We find many phosphoproteins in T and B cell signaling pathways that have been shown to integrate immune signals and regulate cytokine secretion in mast cells [40], interact directly with vav, 85/p110alpha and PLCG1 [41] and regulate TCR-mediated calcium mobilization and Erk activation [42]. GRB2 interaction with LAT and LAX1 observed in breast tumor cells may be an indirect interaction through PLCG1, VAV or PIK3R1. Novel GRB2-interactions (CNAIP and SIT1) with phosphotyrosine located at the ITAM region are critical for activating cytokine promoters [43] as well as viral virus induced mammary tumors [44]. GRB2 interaction with a transmembrane adaptor protein (NTALpY95 and pY136) downstream of FcvarepsilonRI receptor was shown to be important in antigen induced calcium signaling and degranulation in mast cells [45]. Parallel to observations in mantle cell lymphoma (MCL) tyrosine phosphoproteome analysis [46], we see active novel phospho-motifs on several negative regulators of immune signals (LAX1, SHIP1pY556; PILRApY246; IL1RL1pY99 and TNFL6pY258) (Figure 7c). Abnormal expression of calcium channels confer proliferative and survival advantage in cancerous cells [47]. Parallel to this, we observed upregulated GRB2- SPTAN1pY2430 interaction in breast tumor cells. GRB2-immune signaling interactome indicates crosstalk between calcium and cytokine signaling pathways in tumor cells to invoke cellular responses to fight and evade the host immune system in the process of neoplastic transformation (Figure 7c).

**Figure 8:**
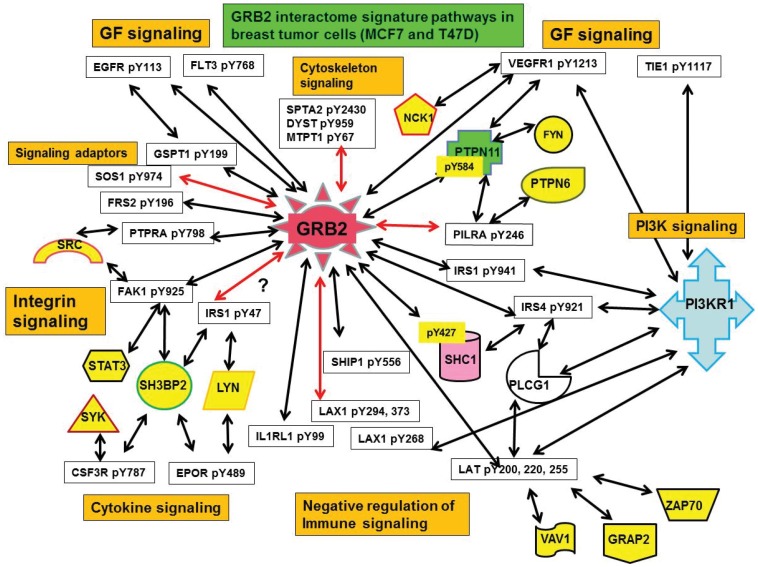
Pathway interactome of upregulated GRB2-pY proteome interactome in breast tumor cells (MCF7 and T47D). The GRB2-pY proteome interactions imply cross-talk between various signaling pathways that are functional to initiate various cellular responses. The GBR2 interactome revealed connections of RTK pathways with both classical GRB2-SOS-RAS cascade with PI3K-IRS1 cascade leading to AKT and ERK activation. The crosstalk between growth factor and cytokine signaling pathways as revealed by major receptors and adaptors and other downstream proteins. Upregulation of negative regulation of immune signaling pathways in tumor cells indicate the preparedness of tumor cells to fight and survive attack from host immune signaling mechanisms. Novel interactions are marked by red arrows.

Tyrosine phosphatases are interesting from the point of RTK regulation in cancer. We found that SHP2pY584 shows very strong interaction with GRB2 in tumor cells (MCF7 and T47D) compared to MCF10A or MDAMB231 (Figure 7d). Consistent with the finding that phosphorylation of SHP-2 on Y279 down-regulates growth factor-induced sustained ERK activation and proliferation through Abl kinase [31], we found GRB2-SHP2pY279 downregulated in breast tumor cells. SHP2pY584-GRB2 interaction is insensitive for Erk activation through PDGF and FGF [48]. Upregulation of SHP2y584-GRB2 interaction in tumor cells suggest that Erk activation is driven through RTKs (EGFR, VEGFR) other than PDGF and FGF to promote cell proliferation and survival. Figure 8 gives the comprehensive upregulated network of GRB2 interactome in ER positive tumor cells (MCF7 and T47D) interconnected to various PTMs of other proteins representing various signaling pathways. The red lines indicate novel interactions not reported so far. The proteins in yellow, pink and green are other adaptor proteins that show interactions with PTMs along with GRB2.

Validation studies to test the association of GRB2 protein with tyrosine phosphoproteins on the peptide chip platform by immunoprecipitation of phosphoprotiens followed by western blotting for GRB2 confirmed that phospho-motif-GRB2 interactions on the chip microarray (*in vitro*) are indeed *in vivo* interactions as corroborated by reports from previous studies. The expression analysis of GRB2 and a few phosphoproteins used in the study showed differences in protein expression and did not reflect changes similar to what we saw in chip assays supported the view that these GRB2 interaction differences might be not only due to expression but might also be due to stoichiometry of phosphorylation in cancer cells.

**Figure 9:**
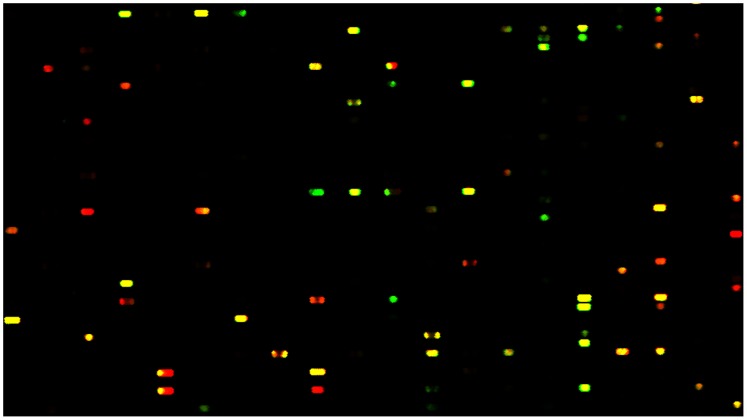
Phosphopeptide microarray analysis of human cardiomyocyte progenitor cells showing detection of multiple proteins (GRB2 and SRC) on the same phosphoprotein-protein interaction complexes. The red spots refer to the presence of GRB2 protein (Rabbit Anti GRB2 with Rabbit anti-IgG conjugated with Alexa 647); The green spots refer to SRC (Mouse Anti SRC with Mouse anti-IgG conjugated with Alexa 594); The yellow spots is the results of merge of GRB2 and SRC images together showing the presence of GRB2 and SRC on the same phosphopeptide-protein complex.

Differential cell signaling networks based on the peptide microarray data in tumor cells that distinguish tumor cells from metastatic cells demonstrate the feasibility of identify protein interaction dynamics that could differentiate neoplastic transformation and metastasis (Figure 9). Phosphopeptides are sensitive and informative probes able to detect endogenous protein signaling complexes and active phospho-motifs from cells tissues and tumor samples. The PPEP array thus establishes a probe-target platform for analysis of protein interactome networks. A major challenge ahead is to refine this technology so that we can use quantitative estimation of binding affinity with a phosphotyrosine-motif as an indicator of stoichiometry of phosphorylation and protein expression.

We hypothesized that high concentration of phospho-peptide probes (1 to 4 uM) compared to the significantly lower concentration of a phospho-motif *in vivo* (by at least 100 to 10000 fold) is sufficient to trap phosphoprotein-protein interactions at the respective phosphopeptide probe on the chip. To determine if the phosphopeptide probes on the chip could pull down respective phosphoprotein-protein complexes from cell lysate as indicated by phosphopeptide array results, we performed based pull down assays from cell lysate using biotin labeled 6-mer phosphopeptide with a 6-carbon linker that separated labeled biotin and the peptide. We were unable to pull down protein complexes specific for the phosphopeptide probes (data not shown) because the linker that separates phosphopeptide from the glass surface on the chip is very long (with two 6 carbon AHX linkers followed by asparagine and 4 layers of lysine) compared to what we used in the cell assays. We are currently working on optimizing the design of phosphopeptides to be used in pull down assays.

Minimal quantities of sample (100 ug of total protein from cells, tumor or serum) and detection reagents (0.5 to 1 uL of high affinity antibody/chip/detection) are the major strengths of the µParaflo® PepArray technology that will enable screening of clinical samples with limited quantities. The total reaction volume of this microfluidic surface is only 10 uL but it can detect 4,000 to 30,000 different phosphopeptide interactions in a single assay. The technology is sensitive enough to identify protein interactions from sample amounts as low as 300 ug of total proteins (Data not shown) without losing the dynamic range of signal detection from as low as 500 to the level of saturation (65K). Establishing disease signaling pathway focused microarray scan stations for sample (blood/tissue/cell) analysis from cancer patients will be a significant step in clinical diagnostics for patient stratification and determination of optimal treatment strategies.

In order to see if we could detect two different proteins from protein complexes bound to the phosphopeptide probes, we performed a total protein binding assay on chip and used two different species of antibodies (anti-rabbit GRB2 and anti-mouse SRC) and the signals were detected using respective rabbit and mouse secondary antibodies conjugated with Alexa dyes (Alexa 647 and 594) showing maximal absorption at two different wavelengths. The superimposed images of GRB2 and SRC binding images showed the presence of GRB2 and SRC proteins on protein complexes either independently (red spots indicate GRB2 binding and green spots indicate SRC binding) or together (yellow spots indicate the presence of GRB2 and SRC together). The results indicated the possibility of identifying more than one protein in the same interactome complex by using two different protein specific antibodies using duplicate cell lysate samples on two independent chips of the same peptide design (Figure 9). The results reveal another dimension of this technology to detect proteins of interest from unknown protein complexes that interact with specific tyrosine phosphoproteins. Availability of quality controlled non cross-reacting antibodies of different species for different proteins could enable detect of 4 different proteins (the recent advanced versions of microarray scanners can read signals from 4 different wavelengths) from a single assay.

Tremendous advancements have been made for the global analysis of proteomes with high resolution mass spectrometry combined with sample preparation techniques requiring as low as 1–2 ug of total proteins for identifying 2000 proteins (e.g. FASP) as alternatives to both “in-gel” and “in-solution” digestion of proteins [49], [50]. Many laboratories still use conventional mass spectrometry analysis of proteins from CBB stained SDS-PAGE gel pieces as the starting material for PTM identification which requires atleast a few milligrams of total protein [51], [52], [53]. At present the PhosphoScan technology (Cell Signaling Technology) and SILAC based methods (pioneered by Mann from Max Planck Institute and others) which are non-gel based “in-solution” methods of sample preparation are the best known technologies for the large scale global analysis of PTMs. But the requirement of huge amounts of protein (20–40 mg of total protein) requirements, irrespective of any existing methodology, is a major impediment for the application of advanced mass spectrometry techniques to analyze phosphoproteome on a global scale from clinical samples that are of limited quantity. The technical difficulties associated with tyrosine phosphorylation identiifications by mass spectrometry is mainly attributed to lower relative abundance of phosphoproteins, low stoichiometry of tyrosine phosphorylation and the labile nature of pY events during chemical manipulations required for mass spectrometry analysis [54]. Given the difficulties associated with tyrosine phosphosite identification by mass spectrometry and the amounts of total protein required, a simple µParaflo® PepArray based phosphopeptide-protein binding assay using 300 to 500 ug of total proteins as supported by the results from our present work, could potentially be used not only to identify tyrosine phosphorylation of a putative or a previously identified protein motif, but also detect proteins interacting with that motif to reveal interaction networks that are initiated from that nodal point. Using multiple antibodies, it is possible to detect multiple proteins in the protein complex bound to a phosphopeptide probe. Integration of this chip platform with mass spectrometry would enable us to recover and detect all the endogenous complex proteins trapped on phospho-peptides.

An ongoing project on phosphopeptide microarray analysis of human ventricular cardiomyocytes treated with various cancer drugs to simulate a chemotherapy induced cardiotoxicity response revealed time-dose dependent upregulation and down regulation of endogenous phosphoprotein-GRB2 interactions (Krishnamoorthy and Gao unpublished). Similar signature protein profiles of drug response can be used as companion diagnostics for validation TKI drug specificity and identification of off target proteins activated by the drug candidate.

## Supporting Information

Table S1 **Layout file of high affinity phosphopeptides selected from recombinant protein binding assays that are substrates for SH2 domain containing proteins for cell lysate assay.**
(XLSX)

Table S2 **Layout file of phosphopeptide array of RTK pathway proteins for cell lysate assay.**
(XLS)

Table S3 **KEGG pathway analysis of the source peptide probes used in SH2 domain binding assays.**
(XLS)

Table S4 **Data of selected phosphopeptide probes (PPEPs) from the peptide microarray assay of recombinant SH2 domain protein binding.**
(XLS)

Table S5 **Molecular classification and annotation of selected phosphomotifs based on respective phosphopeptide data showing strong interaction with endogenous GRB2 from breast normal and cancer cells.**
(XLS)

Table S6 **Processed data of GRB2-RTK phosphopeptide microarray consisting of 643 pY sites on for MCF10A, MCF7, T47D and MDA-MB231 cell lines.**
(XLS)

Table S7 **GRB2 binding data for all the pY motifs for selected proteins from RTK pathway proteome.**
(XLS)
