# Correction: A Novel Phosphopeptide Microarray Based Interactome Map in Breast Cancer Cells Reveals Phosphoprotein-GRB2 Cell Signaling Networks

**DOI:** 10.1371/annotation/a05f0793-f1af-409d-984e-79444877aec3

**Published:** 2013-09-19

**Authors:** Srinivasan Krishnamoorthy, Zhonghua Liu, Ailing Hong, Ruijuan Zhu, Haosi Chen, Tongbin Li, Xiaochuan Zhou, Xiaolian Gao

A funding organization for the seventh and eighth authors was incorrectly omitted from the Funding Statement. The Funding Statement should read: The work is supported by R33 grant from NCI (4R33CA126209) through Clinical Proteomic Technologies for Cancer(CPTC)to XG and XZ. 

